# Impact of involving the community in entomological surveillance of Triatoma infestans (Klug, 1834) (Hemiptera, Triatominae) vectorial control

**DOI:** 10.1186/s13071-021-04608-6

**Published:** 2021-02-05

**Authors:** L. Abrahan, M. J. Cavallo, I. Amelotti

**Affiliations:** 1 Centro Regional de Investigaciones Científicas y Transferencia Tecnológica de La Rioja (CRILAR), UNLAR, SEGEMAR, UNCa, CONICET, Entre Ríos y Mendoza s/n, Anillaco (5301), La Rioja, Provincia de La Rioja Argentina; 2 Centro de Investigaciones y Transferencias de Catamarca (CITCA)-CONICET-UNCA, San Fernando del Valle de Catamarca, Catamarca, Argentina; 3 Universidad Nacional de La Rioja (UNLAR), La Rioja, Argentina

**Keywords:** Triatomines, Kissing bugs, Chagas disease, Community participation

## Abstract

**Background:**

Vectorial transmission is the principal path of infection by *Trypanosoma cruzi*, the parasite that causes Chagas disease. In Argentina, *Triatoma infestans* is the principal vector; therefore, vector control is the main strategy for the prevention of this illness. The Provincial Program of Chagas La Rioja (PPCHLR) carries out entomological evaluation of domiciliary units (DUs) and spraying of those where *T. infestans* is found. The lack of government funds has led to low visitation frequency by the PPCHLR, especially in areas with a low infestation rate, which are not prioritized. Therefore, seeking possible alternatives to complement control activities is necessary. Involving householders in entomological evaluation could be a control alternative. The major objective was to determine the cost of entomological evaluation with and without community participation.

**Methods:**

For entomological evaluation without community participation, PPCHLR data collected in February 2017 over 359 DUs of the Castro Barros Department (CBD) were used. For entomological evaluation with community participation, 434 DUs of the same department were selected in November 2017. Each householder was trained in collecting insects, which were kept in labeled plastic bags, recovered after 2 weeks, and analyzed in the laboratory for the presence of *T. cruzi*. Using householders' collection data, a spatial scan statistic was used to detect clusters of different *T. infestans* infestations. Entomological evaluation costs with and without community participation related to the numbers of DUs visited, DUs evaluated, and DUs sprayed were calculated and compared between methodologies. In addition, the number of DUs evaluated of the DUs visited was compared.

**Results:**

According to the results, the triatomines did not show evidence of *T. cruzi* infection. Spatial analysis detected heterogeneity of *T. infestans* infestation in the area. Costs related to the DUs visited, evaluated, and sprayed were lower with community participation (*p* < 0.05). In addition, more DUs were evaluated in relation to those visited and a greater surface area was covered with community participation.

**Conclusion:**

Participation of the community in the infestation survey is an efficient complement to vertical control, allowing the spraying to be focused on infested houses and thus reducing the PPCHLR's costs and intervention times.
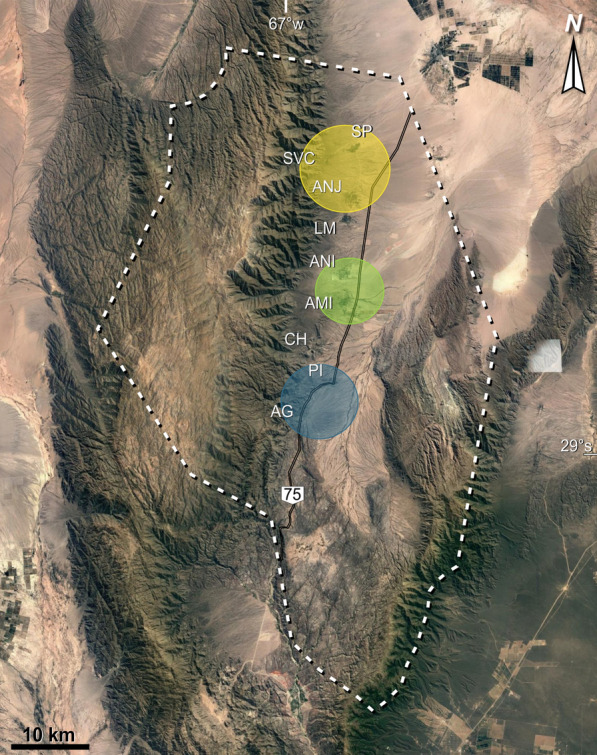

## Background

Chagas disease continues to be an important public health problem in Latin America where an estimated 6 to 7 million people have been infected with the *Trypanosoma cruzi* (Chagas, 1909) (Kinetoplastida, Trypanosomatidae) parasite, the causative agent of this disease [1]. In Argentina, it is assumed that 1 to 3 million people could have this disease, although there are currently no official data on the number of people infected or at risk of *T. cruzi* infection [2].

This parasite can be transmitted through different ways, but mainly through vector transmission, that is, by contact with feces of infected triatomines [1], so the vectorial control of this infestation is the central strategy for the prevention of the illness.

In Argentina, *Triatoma infestans* (Klug, 1834) (Hemiptera, Triatominae) is the triatomine species with the greatest epidemiological importance, given its ability to inhabit inside and on the periphery of houses. In this country, Chagas disease vector control is focused on *T. infestans* infestation.

La Rioja province is endemic for Chagas disease and considered of medium risk for the transmission of this disease by *T. infestans* [3]. The Provincial Program of Chagas La Rioja (PPCHLR) works on entomological evaluation and insecticide spraying in positive houses to eliminate *T. infestans* infestation. When vector control actions are carried out in a sustained and committed way over time, triatomines' presence in houses is reduced and consequently the risk of vector transmission decreases. However, in areas where the infestation is reduced, a paradox occurs as these areas lose surveillance priority and are visited less frequently, and their chemical treatments are postponed. This misconception produces a huge setback in achieving the main objective, which is vectorial transmission interruption. Another factor is that the householder's claims are not considered, so the houses where the PPCHLR does not find *T. infestans* do not receive treatment.

For entomological evaluation of houses, the PPCHLR staff moves from the capital city to the different departments and returns to them depending on political decisions about economic resource utilization. Therefore, the PPCHLR focuses surveillance on departments with high infestation rates.

The Castro Barros Department (CBD) has had a low frequency of vector control interventions, that is, every 3 or 4 years. This situation is a consequence of the lower *T. infestans* infestation rate in relation to other areas that have been a priority for the PPCHLR, such as the San Martin Department [4, 5]. It is known that longer intervention intervals increase the risk of recovery of *T. infestans* populations [6]. Simultaneously, the community demands vector control activities because of the frequent *T. infestans* presence as it is impossible to consolidate control and surveillance actions with vertical strategy methods in extended rural areas [7].

A theoretical vertical vector control model would be annual interventions by specialized technicians who evaluate and spray houses [8]. However, the logistical capacity does not exist in La Rioja Province. Given the actual situation, the advantages and disadvantages of maintaining only vertical PPCHLR interventions in low infestation areas need to be re-evaluated. Therefore, in this area, community participation in entomological surveillance would be an essential tool given this complex scenario, but the cost of this type of control activity needs to be evaluated.

Several authors have reported the advantages of more active community participation by having householders collect triatomines in their homes in response to reports on the presence and control of *T. infestans* populations (e.g., [9–11]). The use of this method allows extending the search time and obtaining infestation data in the houses, which are often not possible because of the limited time allocated to them [11].

Householder collection of triatomines may be more sensitive than active searches in some areas [11–14], especially when the infestation is of low density [15].

In addition, triatomines were reported more by householders than by active searches [11]. Several authors have mentioned that community participation should be considered instead of active searches to detect *T. infestans* infestation foci, although recognizing that the effect is more pronounced in dwellings than in their surrounding structures [13–16].

Community-based vector control is the most cost-effective alternative in rural areas with limited resources [14, 17]. In a previous study, our work team concluded that real field data on the costs of different control methods were needed to complete the entomological surveillance analysis [13].

This study originated within a larger project in response to the community's request to our research team, which resides in CBD. The main objectives of this work were to determine the cost of vectorial control activities with and without community participation and to analyze the spatial distribution of *T. infestans* infestation in the study area.

Although part of the study aimed to reduce costs by involving the community in entomological surveillance, the main objective was to quantify the costs of each methodology with real field values to determine the magnitude of this difference in detail. It is important to clarify that the work does not compare the methodologies' sensitivities or infestation detection differences among the field samples.

## Methods

### Study area

CBD is located to the northeast of La Rioja Province, Argentina. Its departmental head is the Aminga locality, 95 km from the capital city. It is located in the biogeographic region of Monte Desert. The population density is around three inhabitants/km^2^. It is a rural population, concentrated in ten localities that function as an oasis due to the availability of surface water. The total population of CBD is 4268 inhabitants [18].

Domestic infestation from 2009 to 2013 ranged from 1.18% to 9.87%. In 2013, the latest departmental intervention was carried out by the PPCHLR without community participation (PPCHLR unpublished data).

Each intradomicile (ID) with its peridomestic structures (PDs) was defined as a domiciliary unit (DU). A DU was recorded as “infested or positive” when at least one *T. infestans* individual was found in the DU.

### Entomological evaluation without community participation

In February 2017, 359 DUs localized in Aminga (one of ten localities of the department) were visited for entomological evaluation, carried out by 20 PPCHLR technicians. A dislocating agent (tetramethrin 2%) was used in the search, which was interrupted when an insect was found or until an hour of capture effort had been completed (hour/person method). The infestation status and treatment of each georeferenced DU were registered. This type of control was defined as vertical intervention.

An evaluated DU was defined as a DU in which the householder was present and approved entomological evaluation, while a DU visited was defined as all DUs, including those in which the householder was not present at the time of entomological evaluation carried out by PPCHLR technicians.

### Entomological evaluation by community participation

**Fig. 1.**
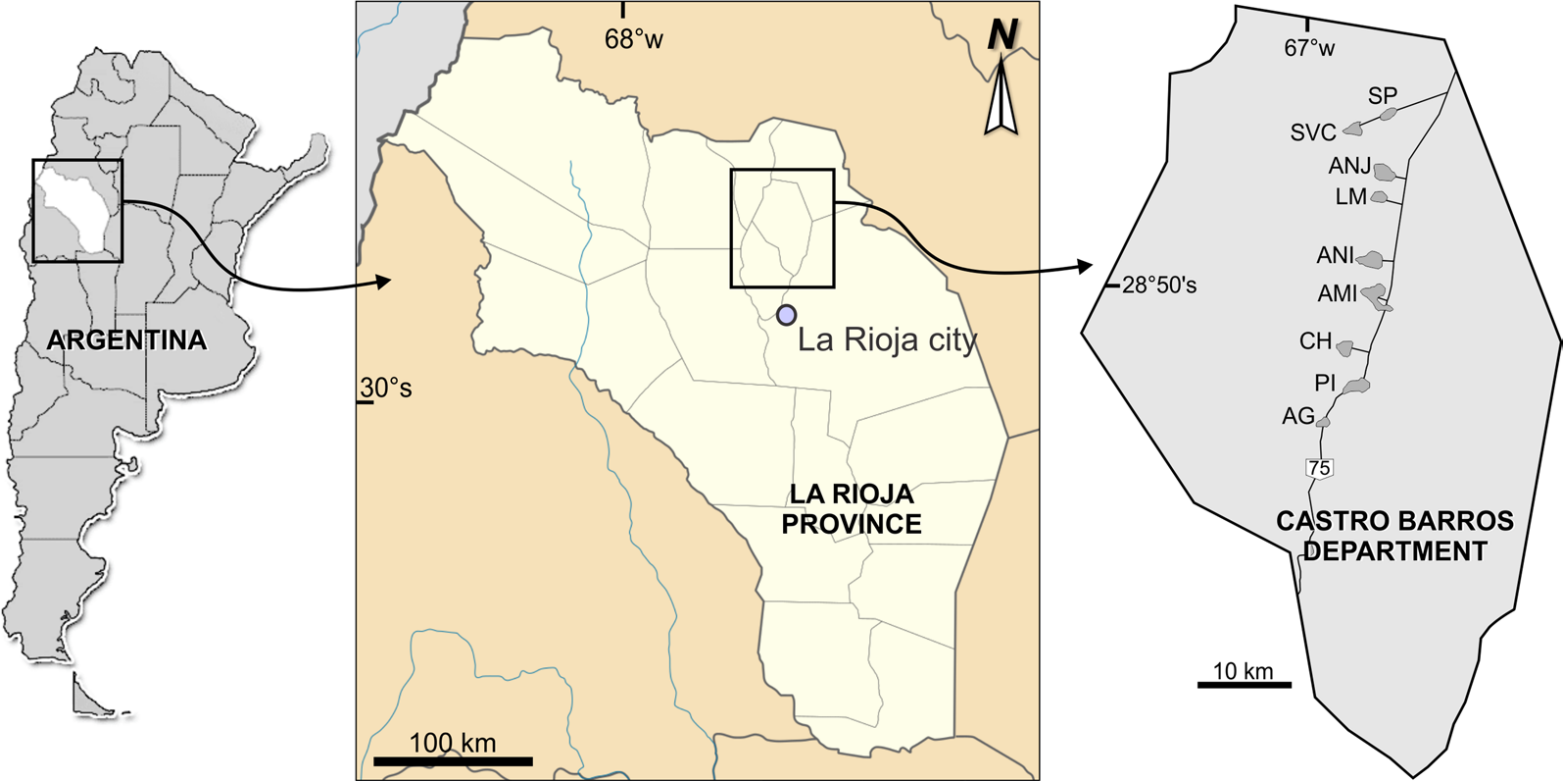
Geographical localization of Castro Barros Department and localities evaluated with community participation in December 2017. *SP *San Pedro, *SVC* Santa Vera Cruz, *ANJ* Anjullón,* LM* Los Molinos, A*NI* Anillaco, *AMI* Aminga, *CH* Chuquis, *PI* Pinchas, *AG* Agua Blanca

In December 2017, 434 DUs from 9 localities in CBD (Fig. 1) were visited, and *T. infestans* infestation was evaluated by the householders [13].

The samples were selected in three steps. First, the total number of DUs in each locality was counted. Second, the minimum DU number was estimated to guarantee coverage of at least 20% of the total DU number in each locality. Finally, DU samples from each locality were included considering two factors: the best representation of all points in the locality and the householder's disposition and interest in participating in this study (Table 1).

**Table 1:** Description of the study area for each locality

Locality	Total number of DUs^a^	Inhabitant number^b^	Altitude (masl)	Number of DUs visited	Coverage (%)^c^
San Pedro	145	298	1507	16	11
Santa Vera Cruz	79	123	1323	16	20
Anjullon	242	418	1294	42	17.36
Los Molinos	166	244	1254	35	21
Anillaco	678	1573	1325	110	16.22
Aminga	359	833	1275	99	27
Chuquis	157	236	1323	44	28.03
Pinchas	262	390	1351	55	20.99
Agua Blanca	30	68	1470	17	56.7

^a^Local hospital database

^b^INDEC (Instituto Nacional de Estadística y Censos 2010)

^c^Coverage is calculated as the number of DUs visited over the DU total number

Inhabitants of the selected DUs received a detailed explanation of the study and were invited to participate. Inhabitants that accepted the invitation were trained in triatomine identification, places to search, and careful collection methods to avoid the risk of accidental infection. Each participating family received plastic bags labeled with the DU identification code for different ecotopes. A registry was made with the participating families and the characteristics of their DU. The householders’ collection period was 2 weeks in November and December 2017 (from delivery to plastic bag collection). Simultaneously, health agents from local hospitals worked alongside the householders.

The collection bags were transferred to the laboratory where the species, gender, and developmental stage [19] were determined, and *T. cruzi* detection was performed using rectal material. The number of *T. infestans* according to gender and sex was quantified for each locality. For *T. cruzi* analysis, the fresh fecal samples were examined in a drop of physiological solution using an optical microscope at 400× magnification for approximately 15 min (25 fields). The PPCHLR was notified about the presence of *T. infestans* registered in DUs so they could carry out pyrethroid insecticide treatment.

A visited DU was defined as one in which the householder had been given bags for insect collection.

An evaluated DU was defined as one in which the householder gave us bags with or without material. A closed DU was defined as one in which the householder was not present at the time of bag collection. The difference between DUs visited and DUs evaluated was that some residents received a collection bag but were not in their home at the sample recovery time.

### Costs with and without community participation

To determine the cost of vectorial control activities with and without community participation, CBD campaign countable information given by PPCHLR was used (February and December 2017).

The cost without community participation was only constituted by the ‘expenses’ (fuel and travel wage) incurred by the PPCHLR.

When householders participated, the cost comprised the sum of the expenses incurred by PPCHLR and inputs used (brochures, gloves, and fuel) for traveling to the study area to train householders or to collect the bugs.

The costs of the DU visited number over DU evaluated number and in relation to the DU sprayed number were calculated for each methodology.

The insecticide and spraying machine costs were not considered since they were the same for both methodologies. In both cases, the supplies to carry out spraying were provided by the National Chagas Program and did not involve an additional cost for the PPCHLR.

### Climatic Variables

To verify the climatic conditions in the study area and based on equipment availability, three data loggers (HOBO U10/003 Onset Computer Corp, Bourne, MA, USA) were respectively placed in the Pinchas, Anillaco, and Santa Vera Cruz localities (Fig. 1). Temperature (°C) and relative humidity (%) were recorded at 15-min intervals between 7:00 p.m. and 10:30 p.m., corresponding to the moment of peak active dispersion of *T. infestans* [20–22].

### Data analysis

The percentage of infested DUs was calculated over the total evaluated DUs by locality.

For cost analysis, data were compared between methodologies using chi-square of the Infostat program [23].

A spatial scan statistic with a Poisson model was used to detect clusters (geographically aggregated groups of localities with higher or lower infestation compared with the regional average). Locality was the analysis unit. Analysis was performed using SaTScan v. 9.4.4 [24].

Climatic variables were compared with a non-parametric Kruskal-Wallis test using the Infostat program [23]. The present study was focused on environmental variables because in a previous work the cleaning degree, peridomestic area, and dwelling typology were not associated with *T. infestans* infestation [25].

## Results

In February 2017, DU infestation was 8.26% (1/109 ID and 8/109 PD), and all DUs were sprayed.

In December 2017, 81.6% of DUs were evaluated (354/434) by householders because 18.4% of DUs were closed. The general infestation by *T. infestans* in the study area was 13.8%, varying between 0 and 50% among localities (Table 2). Of the total DUs with presence of *T. infestans*, 80% (39/49) were registered within the ID, and only ten DUs reported having *T. infestans* in PD. A total of 97.4% positive ID (38/39) and 40% positive PD (4/10) were sprayed with pyrethroid insecticide by PPCHLR technicians.

**Table 2:** Infestation by *T. infestans* obtained by householder collection in Castro Barros Department, La Rioja

Locality	DU evaluated	DU closed^a^	DU infestation% (CI 95)	Number of IDs with *T. infestans* presence^b^	Number of PDs with T. infestans presence^c^
San Pedro	12	4	8.33 (0.44–40.25)	1	0
Santa Vera Cruz	13	3	0 (0–28.34)	0	0
Anjullon	36	6	0 (0–12.00)	0	0
Los Molinos	27	8	14.81 (4.86–34.61)	1	3
Anillaco	84	26	8.33 (3.7–16.95)	7	0
Aminga	73	26	5.48 (1.77–14.16)	4	0
Chuquis	41	3	12.19 (4.58–27.00)	4	1
Pinchas	52	3	38.46 (2.56–52.99)	15	5
Agua Blanca	16	1	50 (27.99–72.00)	7	1

^a^DU closed at time of bag collection

^b^ID: Intradomicile

^c^PD: Peridomicile

Householders collected 79 specimens of *T. infestans* (Additional file 1: Figure S1), 52 individuals in IDs and 27 in PDs, none with presence of *T. cruzi* infection. The number of *T. infestans* collected in localities could not be compared because collection time per householder was not standardized.

When the costs were compared between methodologies (Table 3), data showed a reduction in costs related to DU visited (*χ*^2^ = 4.57, *p* < 0.0325), to those evaluated (*χ*^2^ = 24.64, *p* < 0.0001), and to those sprayed (*χ*^2^ = 13.22, *p* < 0.0003) with community participation. Moreover, more DUs were evaluated in relation to those visited (*χ*^2^ = 23.43, *p* < 0.0001) and a larger surface area was covered (163.9 vs. 0.8 km²) with community participation.

**Table 3:** Comparisons of indicators between methodologies in Castro Barros Department during 2017

Variables	Entomological evaluation by PPCHLR, without community participation (Feb)^a^	Entomological evaluation with community participation (Dec)^b^
Number of DUs visited	359	434
Number of DUs evaluated	109	354
Number of DUs sprayed after evaluation	57	43
Surface area evaluated (km^2^)	0.796	163.89
Cost	3809.90	1323.71
Cost related to the number of DUs visited	10.61*	3.05*
Cost related to the number of DUs evaluated	34.95**	3.74**
Cost related to the number of DUs sprayed	66.84***	30.78***

^*^χ^2^ = 4.57, *p* < 0.0325; **χ^2^ = 24.64, *p* < 0.0001; ***χ^2^ = 13.22, *p *< 0.0003

^a^Cost without community participation was constituted by the ‘expenses’ (fuel and travel wages) incurred by PPCHLR

^b^Cost with community participation was constituted by the sum of the expenses incurred by PPCHLR and inputs used (brochures, gloves, and fuel) for traveling to the study area to train householders or to collect bugs

**Fig. 2:**
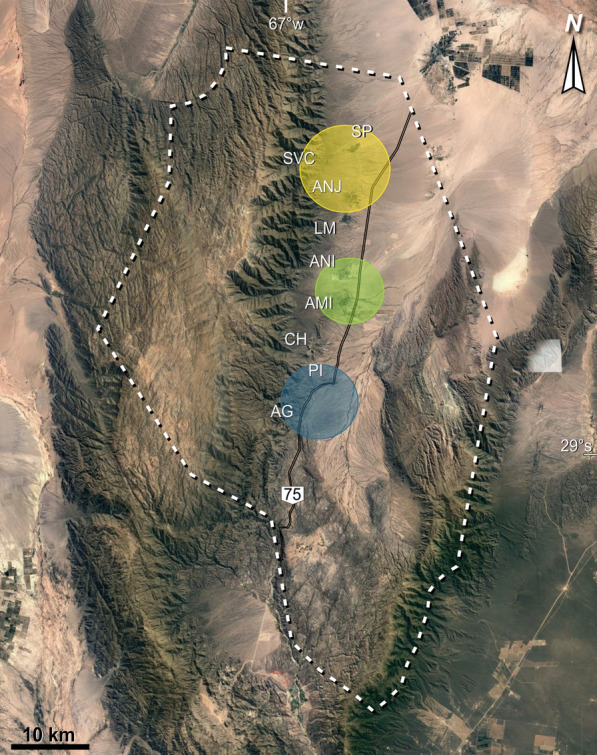
Clusters of localities in the Castro Barros Department with high and low *T. infestans* infestation rates. Entomological evaluation with community participation in December 2017. Each circle represents a cluster area and group localities with similar *T. infestans* infestation rates. North Zone cluster (low infestation = 0.02%). Center Zone cluster (low infestation = 0.07%). South Zone cluster (high infestation = 39.7%). *SP *San Pedro, *SVC* Santa Vera Cruz, *ANJ* Anjullón,* LM* Los Molinos, A*NI* Anillaco, *AMI* Aminga, *CH* Chuquis, *PI* Pinchas, *AG* Agua Blanca

The spatial analysis allowed detection of differences in infestation with respect to the average area. Three clusters were identified in the area (Fig. 2). The first cluster, called the North Zone, presented an infestation rate of 0.02%, which is less than the average in the area (relative risk = 0.1; *p* = 0.02), and covered three localities, San Pedro, Santa Vera Cruz, and Anjullón, with 61 DUs and a radius of 6.33 km centered at − 28.66°S, − 66.92°W. The second group of localities comprised the Center Zone cluster, with an infestation rate of 0.07%, also lower than expected (relative risk = 0.37; *p* = 0.04), with two localities, Aminga and Anillaco, with 157 DUs and a 4.68 km radius centered at -28.85° S, -66.93° W. The third cluster, called the South Zone, had an infestation rate of 39.7%, which was higher than expected (relative risk = 5.4; *p* < 0.001). It covered two localities, Agua Blanca and Pinchas, with 68 DUs and a 4.88 km radius, centered at -28.96° S, -66.99°W.

As possible factors that could influence zonal infestation differences, temperature, and relative humidity were compared among clusters. The South Zone had higher temperatures than the Center Zone, which in turn had higher temperatures than the North Zone (H: 96.73, gl: 2, *p* < 0.0001). Concerning relative humidity, the South Zone had lower humidity than the Center Zone, which in turn showed lower humidity than the North Zone (H: 59.51, gl: 2, *p* < 0.0001). Table 4 shows the median temperature and relative humidity.

**Table 4:** Median of temperature (°C) and relative humidity (%) for each cluster (quartiles 1 and 3)

Cluster	Variable	Median	Quartile 1	Quartile 3
North Zone	Temperature	22.33	20.42	24.84
	Relative humidity	52.53	42.67	62.44
Center Zone	Temperature	26.68	25.42	28.16
	Relative humidity	40.03	34.01	45.94
South Zone	Temperature	28.06	26.98	29.45
	Relative humidity	35.82	30.64	38.38

## Discussion

In this work, the impact of incorporating community participation in areas with low domestic infestation, which in general is neither a focus of study nor a priority when applying vector control actions, is analyzed. The low frequency of a vertical program cannot meet demand in areas where the infestation risk is known to be low [5, 7], causing domestic vector persistence to continue and allowing population recovery between spraying cycles [6, 26]. Incorporating participatory approaches against vector-borne diseases has been shown to be important for control program sustainability [9, 13, 17, 26–30]. Periodic inspection allows early detection of new foci of reinfestation in the intradomiciles [11, 14, 31]. However, a bio-eco-social approach alone does not always reduce the infestation [32]. In addition, one of the main criticisms of the incorporation of community participation in health programs refers to the process and place given to the community in the decision-making process [8]. Different people bring different assessments to a situation and these must be taken into account [33]. In this study, community intervention was the focus in the surveillance phase to guarantee early triatomine detection. Furthermore, an active and positive attitude was promoted in the local population, and the householders were able to voice their doubts about the transmission and prevention of Chagas disease.

In this work, using field data collected in the same year and without modeling for indirectly estimated variables, two intervention types were compared, showing that costs related to DUs visited, evaluated, and sprayed were lowered with community participation. In addition, more DUs were evaluated and a larger surface area was covered with community participation. Many works have shown a cost decrease when a community collaborates in surveillance [11, 17, 30, 34–37], although with completely different approaches that do not allow a direct comparison with our data. Some studies have focused on vectorial control costs; for example, in Mexico, the cost to evaluate a domicile entomologically to detect *T. dimidiata* (Latreille, 1811) was US$70 for an infested house by carrying out an active search and only US$10 when householders were involved [11]. Also, in Santiago del Estero (Argentina), a very complete analysis was carried out considering community intervention, and the cost-effectiveness was estimated in the attack phase where householders sprayed their own houses [17]. These latter results are not comparable to our data since our focus was only on entomological surveillance and spraying was only carried out by specialized personnel.

In La Rioja Province, it is assumed that a house should be sprayed when PPCHLR technicians corroborate the presence of *T. infestans*. PPCHLR searches are carried out during the day; however, the householders can carry out searches during both the day and night. In this case, the probability of finding dispersants is higher because *T. infestans*' peak activity occurs between 7 and 10 p.m. [20]. Our results showed that most of the insects collected by householders were found in IDs (52/79), of which 13.5% (7/52) were found on external walls or lights or in the mosquito netting, so they were assumed to be dispersants from other sources. Therefore, it is important to establish an appropriate response to each *T. infestans* collected by a householder. Especially female *T. infestans* represent a particular epidemiological risk as colonizers of houses, justifying a control intervention. Each fertilized female can lay 100–600 eggs in her lifetime [38]. Dispersant females carry numerous eggs within their oviducts to ensure successful colonization of a new habitat [21], so it is important not to postpone control actions. In the case of triatomine dispersant collection, the possibilities of invasion can be reduced by physical protection (such as mosquito netting) [8].

To control circuit function correctly and to avoid "false-positive" reports, we proposed that householders inform to the municipal agents about the presence of *T. infestans* in their houses. This requires that each department count of a municipal referent should verify the presence of this species. If houses are *T. infestans* positive, personnel designated for this purpose should spray them and the surroundings. Although in this particular context our CRILAR medical entomology team participates in a social commitment, it is expected that this activity should be carried out routinely by health staff in the area or the Chagas municipal referent, implying that there would be no extra costs. In this way, technicians' work would be optimized, focusing on spraying positive houses already surveyed by sanitary agents, while reducing travel, wage, and fuel costs for the transfer of PPCHLR personnel to the field. Even in a hypothetical deficient detection situation, it is an advantage if houses reported positive by neighbors are sprayed. These economic resources would be designated to increase the treatment frequency by the PPCHLR in areas of higher infestation. Understanding the variables associated with infestation in the area will help design entomological surveillance implementation [8].

Due to the localities involved, the coverage, capture type, and sampling date were different between methodologies, and infestation rates could not be compared. However, the analyses of infestations using the same methodology, within the same study area on the same date, that is, infestation data obtained with community participation in CBD in December 2017, were comparable among areas and allowed detecting zones with different risks of *T. infestans* infestation.

Heterogeneity in infestation probability is known in the areas of Gran Chaco [4, 5, 13, 34, 39]. In addition, *T. infestans* domestic infestation estimated with community participation allowed detecting a spatially heterogeneous infestation in CBD.

Within this department, the southern zone presented the highest risk of infestation. Heterogeneity in the infestation risk could be associated with climatic conditions because the southern zone presented higher temperature and lower humidity compared to the other areas. These climatic conditions could allow optimal growth of the species, as was observed by other authors [34, 40, 41]. Although the climatic variable ranges in the different zones were within the optimal values, the zone with the highest temperature and lowest humidity provided greater development of *T. infestans* populations. The optimal levels for most triatomines are temperatures of 26–29°C and ≤ 70% relative humidity. When temperatures are higher in this range, insects need greater humidity to prevent dehydration. If the climatic conditions are not wet enough, the danger of dehydration can only be countered by increasing the number of bloodmeals, producing a life-cycle reduction with a population increase [42]. Another factor that could explain zonal differences was the presence of PDs because these provided refuge and feeding sources for triatomines [43]. In the southern zone (Agua Blanca and Pinchas), the presence of *T. infestans* in PDs in the evaluated DUs (6/22) was observed, but not in the northern and central zones (Table 2). These results showed that some factors promote the presence of *T. infestans*, particularly in the southern zone of CBD.

An orderly and efficient entomological surveillance system is necessary in rural areas far from the capital with different degrees of urbanism and PD complexity; otherwise, the feasibility of maintaining successful chemical control diminishes. For example, Los Llanos is a rural area with scattered and abundant houses and PD complexes (more than one corral, chicken coop, or warehouse) [13]. Comparatively, in CBD, the houses are aggregated and close, and PDs are lower and less complex.

This work shown that involving the community in entomological surveillance reduced costs, covered a greater surface area and proportion of DUs evaluated, and encouraged early *T. infestans* detection. It is the first step in stimulating control interventions. However, for this strategy to be effective, municipalities should carry out sustained surveillance work and chemical control interventions to prevent *T. infestans* populations from recovering after an application interval. Therefore, these actions must continue to be encouraged, and the authorities must be committed to providing quick and effective responses to householder demands.

## Conclusion

In this study, we provided important and well-founded data on the costs of entomological surveillance when carried out with community participation to complement actions of vectorial control programs between periods of vertical intervention. Community participation is recommended in low infestation areas where a vertical control strategy and adequate control frequency are difficult. This strategy is efficient in increasing collection coverage, allowing spraying to be focused on infested houses, and thus reducing costs and intervention times by control programs, integrating easily with other health programs.

## Supplementary Information

** Additional file: Figure S1:*** Triatoma infestans* number collected by developmental stage and gender in localities evaluated with community participation.

## Data Availability

The datasets supporting the conclusions of this article are included within the article. Raw data are available from the corresponding author on reasonable request.

## Abbreviations

PPCHLR: Provincial Program of Chagas La Rioja
CBD: Castro Barros Department
DU: Domiciliary unit
ID: Intradomicile
PD: Peridomestic structures
