# Supplementary figures and images for: Impact of involving the community in entomological surveillance of Triatoma infestans (Klug, 1834) (Hemiptera, Triatominae) vectorial control
Source: Parasit Vectors. 2021 Feb 5;14:98. doi: 10.1186/s13071-021-04608-6 (PMC7866874; doi:10.1186/s13071-021-04608-6)

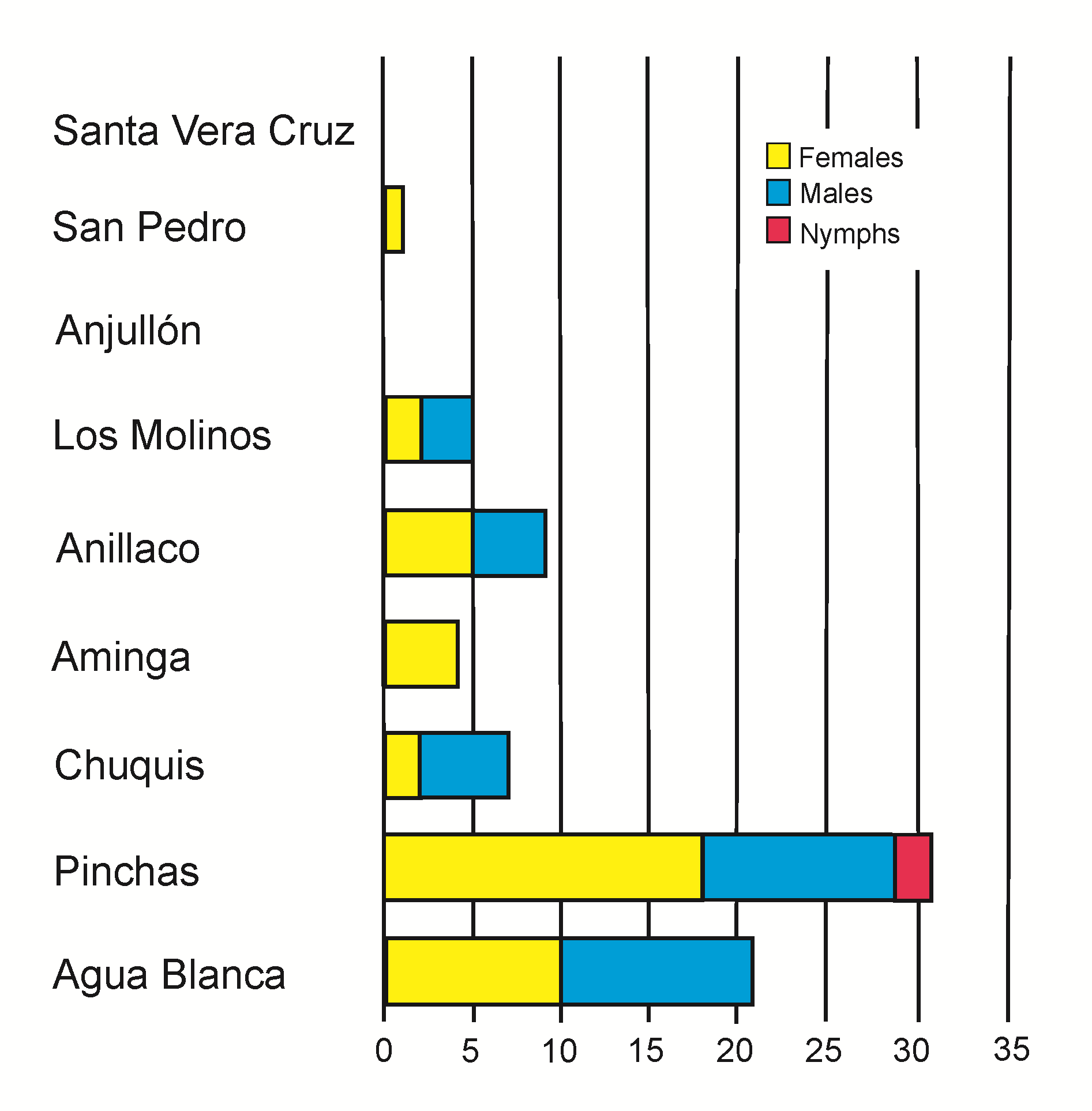

Supplement: Supplementary file 1 — Additional file: Figure S1: Triatoma infestans number collected by developmental stage and gender in localities evaluated with community participation. [file 13071_2021_4608_MOESM1_ESM.tif]
